# Chinese Herbal Medicine Alleviates Thyroidectomy-Induced Cardiopulmonary Exercise Dysfunction in Rats

**DOI:** 10.1155/2020/9415082

**Published:** 2020-03-20

**Authors:** Tai-Yuan Chuang, Chia-Ying Lien, Chih-Hsiang Hsu, Chen-Wen Lu, Chung-Hsin Wu

**Affiliations:** ^1^School of Life Science, National Taiwan Normal University, Taipei, Taiwan; ^2^Department of Athletics, National Taiwan University, Taipei, Taiwan

## Abstract

Hypothyroidism frequently causes cardiopulmonary dysfunction, such as heart failure and respiratory and metabolic deficiencies. This study investigated the effects of Chinese herbal formula B307 on thyroidectomy-induced cardiopulmonary exercise dysfunction in rats. Twenty male rats were equally divided into four groups: negative control with sham treatment, positive control with oral B307 treatment only, thyroidectomy treatment only, and thyroidectomy with B307 posttreatment groups. The feeding dose of B307 was 50 mg/kg per day for 14 days. We examined and then compared the thyroid-stimulating hormone (TSH), free triiodothyronine (T3), free thyroxine (T4), and reactive oxygen species (ROS) from the blood of these four groups. Also, we compared the body weight, neck subcutaneous blood flow, cardiac ejection function, cardiopulmonary exercise function of oxygen consumption (VO_2_), carbon dioxide production (VCO_2_), and respiratory quotient (RQ = VCO_2_/VO_2_) among the four groups. Our results indicated that thyroidectomized rats had significantly decreased body weight, neck subcutaneous blood flow, cardiac ejection function, serum T3 and T4, and VO_2_ and VCO_2_, but had significantly increased ROS and TSH levels and RQ values compared with sham rats (*P* < 0.01–0.05). In addition, thyroidectomized rats receiving oral B307 treatment had significantly increased body weight, neck subcutaneous blood flow, cardiac ejection function, and VO_2_, but significantly decreased ROS and TSH levels and VCO_2_ and RQ values compared with thyroidectomized rats (*P* < 0.01–0.05). We suggest that the B307 could be a protective and beneficial alternative treatment for thyroidectomy-induced cardiopulmonary exercise dysfunction.

## 1. Introduction

Thyroid hormones mainly regulate metabolic activities [1]. Patients usually undergo thyroidectomy surgery in cases of thyroid cancer, hyperthyroidism, or goiter. The surgical removal of all or part of the thyroid gland may cause hypothyroidism, which is caused by thyroid hormone deficiency. A well-established model for the study of hypothyroidism is the use of male rats with near-total thyroidectomy [2]. Thyroidectomy-related hypothyroidism often causes symptoms such as slow heart rate, lower blood pressure, reduced respiration and oxygen consumption, and an increased rate of metabolic diseases and neuropsychiatric disorders [3, 4]. The prevalence of subclinical hypothyroidism is approximately 4% to 10% in Western populations [4, 5]. In Taiwan the prevalence is 4.5% [6, 7]. The prevalence of subclinical hypothyroidism can be as high as 20% in people over 60 years old [8, 9]. Hypothyroidism may affect cardiac function by reducing myocardial contractility [10] and can cause arteriosclerotic and coronary heart disease, leading to higher cardiovascular mortality [3]. Hypothyroidism may also cause dementia and depression, which seriously affect patients' quality of life [11]. Thus, identifying effective treatments for hypothyroidism is crucial.

Chinese herbal medicine is widely used in hyperthyroidism therapy and may reduce the adverse biological effects of thyroxine, slow down the transformation of thyroxine (T4) to triiodothyronine (T3), and modulate the function of the sympathetic nerves or the immune system [12–14]. However, Chinese herbal medicine is rarely mentioned in studies on the treatment of hypothyroidism because it is not commonly used in clinical studies. Hypothyroidism frequently causes heart failure and respiratory and metabolic deficiencies [15]. As our previous studies have suggested, the herbal formula B307 can effectively enhance cardiovascular function and myocardial strength [16, 17]. Ginseng (*Panax ginseng Radix*) and Danshen (*Salviae Miltiorrhizae Radix*) are the main herbal ingredients in B307. Ginseng has antioxidant [18] and anti-inflammatory [19, 20] properties. Danshen can alleviate heart disease and ameliorate the effects of atherosclerosis in humans [21–23] and rodents [24]. In this study, we investigated whether thyroidectomy can induced cardiopulmonary exercise dysfunction. If so, we further investigated whether B307 may be a valuable health supplement for ameliorating thyroidectomy-induced cardiopulmonary dysfunction. To this end, we compared thyroid-stimulating hormone (TSH), T3, T4, and reactive oxygen species (ROS) from the blood and cardiac ejection function between thyroidectomized rats and those without B307 treatment. Furthermore, we also compared cardiopulmonary dysfunction By measuring oxygen consumption (VO_2_), carbon dioxide production (VCO_2_), and respiratory quotient (RQ; VCO_2_/VO_2_) between thyroidectomized rats and those without B307 treatment. We posited that oral B307 treatment could alleviate thyroidectomy-induced cardiopulmonary dysfunction.

## 2. Materials and Methods

### 2.1. Preparation of B307

Similar to our previous studies, B307 was supplied by Sun Ten Pharmaceutical Company.

The main ingredients in B307 are rosmarinic acid, salvianolic acid B, tanshinone IIA from *Salvia miltiorrhiza*, ginsenosides Rb1 from *Panax ginseng*, methylophiopogonanone B from *Liriope spicata*, and schizandrin and gomisin A from *Schisandra chinensis* [16, 17]. Our previous study revealed that the cytotoxicity of B307 is low for SH-SY5Y cells at doses below 50 mg/mL [16]. The B307 feeding dose was 50 mg/kg per day for 14 days. Doses were adjusted according to the weight of each rat. The dosage and administration of B307 in each rat was much lower than the IC_50_ dosage.

### 2.2. Animal Preparation

A total of 20 three-month-old male Sprague–Dawley rats were purchased from BioLASCO Taiwan Co., Ltd. (Yi-Lan, Taiwan). All rats were maintained in an animal facility under specific pathogen-free conditions with a constant temperature of 22°C ± 2°C under a 12 h light/dark cycle; they were given ad libitum access to water and food. All animal experiments were approved by the Institutional Animal Care and Use Committee of our university (Protocol number: NTNU Animal Experiments No. 104022). All rats were randomized into four groups: negative control (sham), positive control with B307 treatment only (CHM), thyroidectomy only (TD), and thyroidectomy with B307 posttreatment (TD + CHM). Before the experiments commenced, all rats were allowed to acclimatize for one week. Before thyroidectomy surgery, rats were anesthetized with isoflurane (Escain; Mylan, Pittsburgh, PA, USA). Then, their thyroid glands were cut out from the tracheal tube. To evaluate the recovery of the wound after surgery, B307 was fed to the TD + CHM rats from the third day after thyroidectomy surgery. The B307 feeding experiment lasted two weeks. We examined the body weight of each rat daily. Also, we observed that the serum TSH of TD rats rapidly increased and then stabilized after seven days. Successful thyroidectomy of the TD and TD + CHM rats was confirmed by macroscopic observation at necropsy.

### 2.3. Subcutaneous Blood Flow Assay

We measured subcutaneous blood flow of rats in the four groups on the last day of the two-week B307 feeding experiment. Following the methods of our previous study [25], the images of subcutaneous blood flow in the chest and abdomen of rats were obtained using a laser Doppler imager (Moor Instruments, Axminster, UK). Then, subcutaneous blood flow was assayed in arbitrary perfusion units by using data acquisition software (MoorFLPI measurement software, Version 3.0; Moor Instruments). For the subcutaneous blood flow assay, we recorded and then averaged three stable consecutive images for each rat.

### 2.4. Cardiovascular Function Assay

We assessed the cardiovascular function of all rats on the last day of the two-week B307 feeding experiment. Following the methods of our previous study [17], a rat was anesthetized with 2% isoflurane gas (Baxter Healthcare, New Providence, RI, USA) and then shaved and coated with a conductive gel (Home Care Technology Co., Ltd., Tainan, Taiwan). After the aforementioned procedures, the rat was placed on a heated working platform for breathing and heartbeat monitoring. Then, a PB406 transducer was applied to obtain left ventricular M-mode images using echocardiography (S-Sharp Corporation, Taiwan). We measured the mitral and aortic blood flow velocity of the anesthetized rat from an apical view. M-mode images of rat hearts enabled the measurement and comparison of cardiovascular function, such as ejection fraction, among the four rat groups. For the echocardiographic assay, we recorded and then averaged three stable consecutive cardiac cycles for each rat.

### 2.5. Cardiopulmonary Function Measurement

We measured O_2_ consumption and CO_2_ production of all rats on the last day of the B307 feeding experiment. By using the AccusCan metabolic measurement system (AccuScan Instruments, Inc., USA), we determined O_2_ consumption and CO_2_ production to assess gas exchange. After acclimation, rats were monitored in the chambers for 24 h. The O_2_ and CO_2_ samples were collected and analyzed every 10 min for each rat from different chambers. Then, data were averaged every hour.

### 2.6. Blood Biochemical Analysis

On the last day of the experiment, all rats were sacrificed and their blood and tissue were collected. A blood sample of each rat was reserved for serum separation, and then the serum was maintained at −20°C for biochemical analysis. The serum samples for each rat were sent to the NCYU Veterinary Laboratory Center to examine TSH, T3, and T4 levels by using a radioimmunoassay kit or enzyme-linked immunosorbent assay. Using an chemiluminescence analyzer (CLA-ID3 CL analyzer; Tohoku Electronic Industrial Co., Ltd., Sendai, Japan), we measured the ROS level of each rat by detecting O_2_^•−^ and H_2_O_2_ activity from the blood sample. The total chemiluminescence count of each rat was calculated by integrating the area under the curve within 10 min.

### 2.7. Statistical Analysis

All data in this study are expressed as mean ± SEM. We used one-way or two-way analysis of variance followed by Student–Newman–Keuls multiple comparison posttest to analyze all data. The data differences were considered statistically significant at *P* values of less than 0.05.

## 3. Results

### 3.1. Oral B307 Treatment Effectively Decreased Abnormal Body Weight Gain in Thyroidectomized Rats

**Figure 1:**
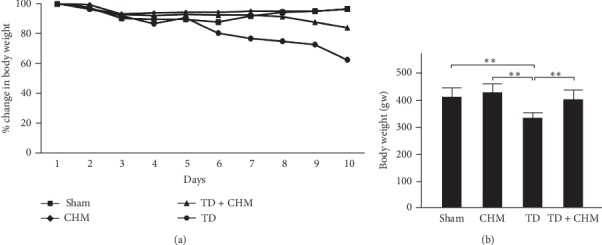
Oral treatment with the herbal formula B307 (CHM) effectively alleviated thyroidectomy-induced body weight gain in rats who underwent thyroidectomy (TD). (a) Changes of average body weight over time. (b) Statistical comparison of quantified body weight among four groups rats of negative control (sham), positive control with B307 treatment only (CHM), thyroidectomy only (TD), and thyroidectomy with B307 posttreatment (TD + CHM). The body weight of TD rats was significantly lower than that of sham rats, whereas the body weight of TD + CHM rats was significantly higher than that of TD rats. The results are shown as mean ± SEM (^*∗∗*^*P* < 0.01, *N* = 5 for each group, two-way ANOVA followed by Student–Newman–Keuls multiple comparison posttest).

The effect of B307 treatment on thyroidectomized rats is shown in Figure 1. Figure 1 illustrates the change in body weight in sham, CHM, TD, and TD + CHM rats. According to case reports, patients with hypothyroidism often gain weight. In contrast to the symptoms in patients with hypothyroidism, we observed gradually decreasing body weight over time in TD rats that underwent thyroidectomy surgery (Figure 1(a)). We observed an obvious body weight decrease in TD rats after the fifth day following thyroidectomy. Following oral treatment with B307, the decreased body weight of TD rats was mitigated (TD + CHM vs. TD, Figure 1(a)). For sham rats (negative control) and CHM rats (positive control), the changes in average body weight over time were quite similar to those observed in TD + CHM rats. The quantified body weights in sham, CHM, TD, and TD + CHM rats are listed in Figure 1(b). We observed that the body weights of TD rats were significantly decreased compared with those of sham, CHM, and TD + CHM rats (Figure 1(b), TD vs. sham, CHM, and TD + CHM, *P* < 0.01). However, no difference was observed in quantified body weight among sham, CHM, and TD + CHM rats (Figure 1(b), TD + CHM vs. CHM vs. sham, *P* > 0.05). The results suggest that oral B307 treatment can alleviate body weight loss for TD rats.

### 3.2. Oral B307 Treatment Effectively Decreased Levels of Thyroid-Stimulating Hormone in the Blood of Thyroidectomized Rats

**Figure 2:**
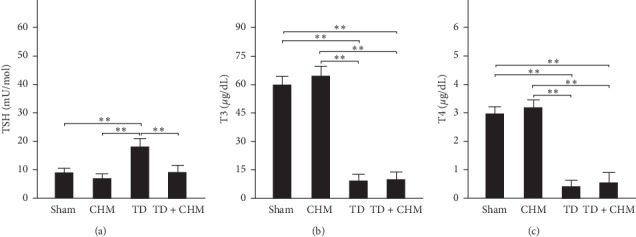
Oral treatment with the herbal formula B307 (CHM) effectively alleviated hypothyroidism-induced abnormal increases in thyroid-stimulating hormone (TSH) in the serum. (a) Statistical comparison of quantified level of serum TSH among sham, CHM, TD, and TD + CHM rats. Levels of serum TSH in TD rats were significantly higher than those in sham, CHM, and TD + CHM rats. (b and c) Statistical comparison of quantified levels of the serum T3 and T4 among sham, CHM, TD, and TD + CHM rats. Levels of serum T3 and T4 of TD and TD + CHM rats were significantly lower than in sham and CHM rats. The results are shown as mean ± SEM (^*∗∗*^*P* < 0.01, *n* = 5 for each group, two-way ANOVA followed by Student–Newman–Keuls multiple comparison posttest).

As described earlier, the complete thyroidectomy of TD rats was dependent on whether serum TSH level increased or not.  Figure 2(a) presents a comparison of serum TSH levels among sham, CHM, TD, and TD + CHM rats by using ELISA. We found that the serum TSH level in TD rats was significantly increased compared with that of sham rats (Figure 2(a), TD vs. sham, *P* < 0.01), and oral treatment of B307 can effectively alleviate the hypothyroidism-induced abnormal increase of TSH serum level (Figure 2(a), TD + CHM vs. TD, *P* < 0.01). We further compared serum T3 and T4 levels among sham, CHM, TD, and TD + CHM rats. We observed that serum T3 and T4 levels in TD and TD + CHM rats were significantly lower than those of sham and CHM rats (Figures 2(b) and 2(c), TD + CHM and TD vs. sham, *P* < 0.01). Different from the serum TSH level change, we observed that oral B307 treatment in TD rats could not alleviate the hypothyroidism-induced abnormal reduction of serum T3 and T4 levels (Figures 2(b) and 2(c), TD + CHM vs. TD, *P* > 0.05).

### 3.3. Oral B307 Treatment Effectively Decreased Blood ROS in Thyroidectomized Rats

**Figure 3:**
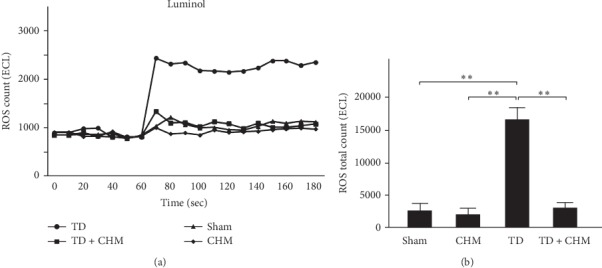
Oral treatment with the herbal formula B307 (CHM) effectively alleviated hypothyroidism-induced increase of reactive oxygen species (ROS) in the blood of TD rats. (a) Change of average blood ROS over time among sham, CHM, TD, and TD + CHM rats. (b) Statistical comparison of quantified levels of total blood ROS among sham, CHM, TD, and TD + CHM rats. Blood ROS levels of TD rats was significantly higher than those of sham rats but significantly lower those TD rats who underwent CHM treatment. The results are shown as mean ± SEM (^*∗∗*^*P* < 0.01, *N* = 5 for each group, two-way ANOVA followed by Student–Newman–Keuls multiple comparison posttest).

To study the effects of B307 on mitochondrial oxidative stress in thyroidectomized rats, we examined and then compared the blood ROS count among sham, CHM, TD, and TD + CHM rats.  Figure 3(a) reveals that only TD rats had markedly increased blood ROS compared with sham, CHM, and TD + CHM rats (TD vs. sham, CHM and TD + CHM). Quantified blood ROS counts of TD rats increased significantly compared with those in sham and CHM rats (Figure 3(b), TD vs. sham and CHM, *P* < 0.01), whereas quantified blood ROS counts in the TD + CHM rats significantly decreased compared with those in TD rats (TD + CHM vs. TD, *P* < 0.01). In other words, rats may have increased blood ROS following thyroidectomy surgery, and oral treatment of B307 may decrease their blood ROS.

### 3.4. Oral B307 Treatment Effectively Increased Neck Subcutaneous Blood Flow and Cardiac Ejection Function in Thyroidectomized Rats

**Figure 4:**
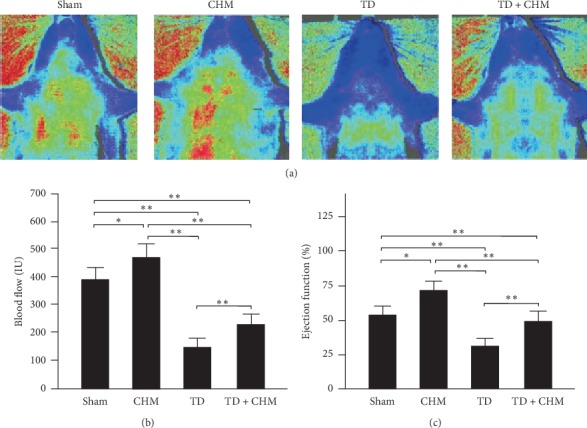
Oral treatment with the herbal formula B307 (CHM) effectively improved neck subcutaneous blood flow and cardiac ejection function in TD rats. (a) Neck subcutaneous microcirculatory flow imaging among sham, CHM, TD, and TD + CHM rats by using moorFLPI laser Doppler imager. Neck subcutaneous blood flow of sham rats with CHM treatment were markedly greater than those sham rats without CHM treatment. While neck subcutaneous blood flow of TD rats with CHM treatment were markedly greater than those TD rats without CHM treatment. (b-c) Statistical comparison of quantified neck subcutaneous blood flow and cardiac ejection function among sham, CHM, TD, and TD + CHM rats. Neck subcutaneous blood flow and cardiac ejection function of TD rats were significantly lower than those of sham rats but significantly higher in TD + CHM rats than in sham rates. The results are shown as mean ± SEM (^*∗∗*^*P* < 0.01, ^*∗*^*P* < 0.05, *N* = 5 for each group, two-way ANOVA followed by Student–Newman–Keuls multiple comparison posttest).

To study the effects of B307 on cardiac function in thyroidectomized rats, we examined and then compared the neck subcutaneous blood flow and cardiac ejection function among sham, CHM, TD, and TD + CHM rats. Using the moorFLPI laser Doppler imager, we observed that CHM rats exhibited increased neck subcutaneous blood flow compared with that in sham rats (Figure 4(a), CHM vs. sham), and TD rats exhibited decreased neck subcutaneous blood flow compared with that in sham rats (TD vs. sham). TD + CHM rats exhibited increased neck subcutaneous blood flow compared with that in TD rats (TD + CHM vs. TD). We quantified neck subcutaneous blood flow among sham, CHM, TD, and TD + CHM rats, and the results are presented in Figure 4(b). We observed that the quantified neck subcutaneous blood flow of TD rats was significantly lower than that of sham rats (Figure 4(b), TD vs. sham, *P* < 0.01). Those rats significantly had increased neck subcutaneous blood flow after they received oral B307 treatment (Figure 4(b), CHM vs. sham, *P* < 0.01). Furthermore, we observed that the neck subcutaneous blood flow of TD + CHM rats was significantly higher than that of TD rats (Figure 4(b), TD + CHM vs. TD, *P* < 0.01). The result suggests that oral B307 treatment may enhance neck subcutaneous blood flow in both sham and TD rats.

We further compared cardiac ejection function among sham, CHM, TD, and TD + CHM rats by using echocardiography. We observed that the quantified cardiac ejection function of rats was significantly increased after they received oral B307 treatment (Figure 4(c), CHM vs. sham, *P* < 0.05) but significantly decreased following thyroidectomy surgery (TD vs. sham, *P* < 0.01). Furthermore, we identified that the quantified cardiac ejection function of TD rats was significantly increased after they received oral B307 treatment (Figure 4(b); TD + CHM vs. TD, *P* < 0.01). These results suggest that oral B307 treatment could effectively enhance cardiac function in both sham and TD rats.

### 3.5. Oral B307 Treatment Effectively Increased VO_2_ but Decreased VCO_2_ with Decreasing RQ Value in Thyroidectomized Rats

**Figure 5:**
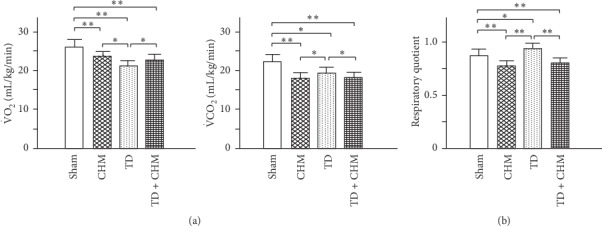
Oral treatment with the herbal formula B307 (CHM) effectively improves oxygen consumption (VO_2_) and decreases carbon dioxide production (VCO_2_) in TD rats. (a) A-Statistical comparison of quantified VO_2_ among sham, CHM, TD, and TD + CHM rats. The VO_2_ of TD rats was significantly lower than in sham rats but significantly higher in TD + CHM rats than in sham rats. B-Statistical comparison of quantified VCO_2_ among sham, CHM, TD, and TD + CHM rats. The VCO_2_ of TD rats was significantly lower than in sham rats and significantly lower in TD + CHM rats than in sham rats. (b) Statistical comparison of quantified respiratory quotient (RQ = VCO_2_/VO_2_) among sham, CHM, TD, and TD + CHM rats. The RQ of TD rats was significantly higher than in sham rats but was significantly lower in TD + CHM rats than in sham rats. The results are shown as mean ± SEM (^*∗∗*^*P* < 0.01, ^*∗*^*P* < 0.05, *n* = 5 for each group, two-way ANOVA followed by Student–Newman–Keuls multiple comparison posttest).

To study the effects of B307 on cardiopulmonary function in thyroidectomized rats, we examined and then compared the oxygen consumption (VO_2_) and carbon dioxide production (VCO_2_) among sham, CHM, TD, and TD + CHM rats. RQ was used to calculate basal metabolic rate (BMR) when estimated from carbon dioxide production. We also examined and then compared the effects of B307 on the RQ values in TD rats. When compared with those of sham rats, we observed that VO_2_, VCO_2_, and RQ values were significantly lower for rats receiving oral B307 treatment (Figure 5, CHM vs. sham, *P* < 0.01). VO_2_ and VCO_2_ were significantly lower, but RQ value was significantly higher for rats that underwent thyroidectomy surgery (TD vs. sham, *P* < 0.05). Furthermore, compared with those of TD rats, we observed that the VO_2_ was significantly higher, but VCO_2_ and RQ values were significantly lower for those TD rats receiving oral B307 treatment (Figure 5, TD + CHM vs. TD, *P* < 0.01–0.05). These results will be interpreted and discussed in a subsequent section.

## 4. Discussion

Thyroid hormones regulate metabolic activities such as growth rate [1], and weight gain is a symptom of hypothyroidism. Individuals with hypothyroidism may experience weight gain because of decreased metabolic rate. Different from symptoms in patients with hypothyroidism, we observed a gradual weight decrease over time in rats that underwent thyroidectomy surgery (Figure 1). Our findings are compatible with those of Yatvin et al. [26], who reported that thyroidectomy may reduce food intake and lead to decreased body weight in rats. They regarded the liver as a major target organ for thyroid hormone action. Thus, hypothyroidism may cause liver dysfunction and alter intestinal handling of cholesterol and bile acids. It is possible that B307 alleviates thyroidectomy-induced digestive disorders.

The causes of hypothyroidism include thyroid gland failure, TSH deficiency, and an inadequate supply of dietary iodine. Primary hypothyroidism indicates defective thyroid synthesis that often causes lower thyroid hormone levels and higher TSH levels in the blood. Secondary hypothyroidism occurs when both TSH levels and thyroid hormone levels are lower, which indicates that the pituitary gland is responsible for low thyroid function. We observed that serum T3 and T4 levels were decreased and serum TSH level was increased in thyroidectomized rats. The finding is compatible with that of a previous study that used thyroidectomy to achieve a state of hypothyroidism. In that study, hypothyroidism was confirmed biochemically in thyroidectomized rats by a significant decrease in serum T3 and T4 levels and a significant increase in serum TSH three weeks after thyroidectomy [27]. The increase in serum TSH level can be explained by a decreased production of serum T3 from the thyroid gland that results in a feedback increase in its secretion by the anterior pituitary gland [28].

As suggested in a review article, it has been reported that reactive oxygen species (ROS) may cause endothelial dysfunction and lead to atherosclerosis and pathogenesis in cardiopulmonary dysfunction [29]. For this reason, we chose ROS as a marker of cardiopulmonary dysfunction. An imbalance exists between free radicals and the antioxidative system in individuals with thyroid dysfunction. A related study indicated that oxidative stress was increased in patients who underwent thyroidectomy surgery and thyroparathyroidectomy [30]. Consistent with previous observations, our results revealed that thyroidectomized rats displayed significantly increased blood ROS levels (Figure 3). A related study also reported that postthyroidectomy hypothyroidism is correlated with oxidative stress, which in turn can be associated with the development of cardiac and vascular dysfunction [31].

Thyroid hormones can also reportedly influence cardiac performance directly and indirectly through changes in peripheral circulation [32]. That study used a unique model of patients who underwent thyroidectomy for thyroid cancer to investigate the cardiac effects of hypothyroidism. For thyroidectomized rats, the reductions of the neck subcutaneous blood flow and cardiac ejection function were associated with increased peripheral resistance and reduced contractility. It is possible that cardiac function is more severely suppressed than oxidative metabolism in thyroidectomized rats. Our results may provide an explanation for the development or worsening of heart failure in thyroidectomized rats. It is reasonable to suggest that CHM B307 could be a protective and beneficial alternative treatment for relieving thyroidectomy-induced cardiac dysfunction.

As suggested in a previous study, cardiopulmonary exercise testing has become an important clinical tool to evaluate exercise capacity and predict outcome in patients with heart failure and other cardiac conditions. It provides assessment of the integrative exercise responses involving the pulmonary, cardiovascular, and skeletal muscle systems. Cardiopulmonary exercise function involves measurements of respiratory oxygen uptake (VO_2_), carbon dioxide production (VCO_2_), and ventilatory measures [33]. Although there are many reports about thyroid hormones and cardiovascular function and hypothyroidism diseases [34], thyroxine as an effective therapeutic strategy for cardiopulmonary dysfunction is still rarely researched and reported. In this study, we found that thyroidectomy may induce cardiopulmonary dysfunction in rats because of decreasing VO_2_, but increasing RQ (please see Figure 5). As suggested from our studies, we have reported that the CHM B307 can be an effective therapeutic strategy for cardiopulmonary dysfunction [16, 17]. Our results showed that CHM 307 can improve cardiopulmonary function because of increasing VO_2_, but decreasing RQ. RQ value is particularly crucial for patients with an impaired respiratory system because abnormally elevated RQ values may increase respiratory rate and reduce tidal volume. This situation may place those patients with impaired respiratory system at risk [34]. Whether thyroxine can be an effective therapeutic strategy for hypothyroidism-induced cardiopulmonary dysfunction in rats need to be further explored.

## 5. Conclusions

CHM B307 therapy in thyroidectomized rats can modulate abnormal elevations of TSH and ROS in the blood, improve cardiac function by enhancing neck subcutaneous blood flow and cardiac ejection function, and alleviate cardiopulmonary dysfunction by increasing VO_2_ but decreasing RQ value. Thus, we suggest that CHM B307 could be a protective and beneficial alternative treatment for relieving thyroidectomy-induced cardiopulmonary dysfunction.

## Data Availability

Blood biochemical analysis, ROS analysis, moorFLPI laser Doppler imager, echocardiography, and AccusCan metabolic measurement system were used to support the findings of this study. The data are included within the article and are available from the corresponding author upon request.

## Abbreviations

CHM: Chinese herbal medicine
ROS: Reactive oxygen species
RQ: Respiratory quotient
T3: Triiodothyronine
T4: Thyroxine
TD: Thyroidectomy
TSH: Thyroid-stimulating hormone
VCO_2_: Carbon dioxide production
VO_2_: Oxygen consumption.
